# Burden and Typing of Rotavirus Group A in Children with Acute Gastroenteritis in Shiraz, Southern Iran

**Published:** 2012-09-30

**Authors:** M Kargar, T Jafarpour, A Najafi

**Affiliations:** 1 Associate Professor of Microbiology, Department of Microbiology, Jahrom Branch, Islamic Azad University, Jahrom, IR Iran; 2 Master science of Microbiology, Department of Microbiology, Jahrom Branch, Islamic Azad University, Jahrom, IR Iran

**Keywords:** Human Rotavirus, Diarrhea, Hospitalized children, G types

## Abstract

**Background:**

Human *Rotavirus* is a significant cause of severe gastroenteritis in infants and young children worldwide. In recent years, *Rotavirus* genotyping by RT-PCR has provided valuable information about the diversity of *Rotavirus*es circulating worldwide. The purpose of the present study is to monitor the prevalence of the different G types of *Rotavirus*es circulating in Shiraz, Southern Iran and detect any uncommon or novel types.

**Methods:**

During the period from December 2007 to November 2008, a total of 138 stool samples were collected from children less than 5 years old who were hospitalized for acute gastroenteritis. *Rotavirus*-associated diarrhea was investigated in fecal specimens with enzyme immunoassays (EIA). *Rotavirus*-positive specimens were typed by the Nested RT-PCR and by using different types of specific primers.

**Results:**

Out of the 138 collected samples, 34.78% (48 cases) tested positive for *Rotavirus*. The frequency of G1, G2 and G4 types was 6.25%, 2.08% and 27.08%, respectively. Mixed and non-typeable infections were detected in 33.34% and 31.25% of hospitalized children with acute diarrhea, respectively. This is the first time mixed *Rotavirus* infections with G1/G3 have been reported in Iran.

**Conclusion:**

The high frequency of *Rotavirus* detection indicates the severity and the burden of *Rotavirus* disease may be able to reduce through the implementation of an effective vaccine and continual surveillance for the detection of *Rotavirus* genotypes circulating in other regions of Iran.

Regarding to the noticeable frequency of non-typeable and mixed infections, it is suggested to use the other specific primers and further studies to detection of other novel and unusual types.

## Introduction

Human *Rotavirus* is the leading cause of severe gastroenteritis in infants and young children worldwide. An estimated 527,000 children less than five years of age die from *Rotavirus* gastroenteritis each year, with >85% of all *Rotavirus*-related deaths occurring in low-income countries of Asia and sub-Saharan Africa.(1) Recent studies have shown that * diarrhea* causes 39% of childhood diarrhea hospitalizations and 5% of all deaths around the world in children less than 5 years old.(2) Nearly every child will infect with *Rotavirus* disease by age 5 years, one in five will require a clinic, one in 50 will be hospitalized, and one in 205 will die from this disease.(3) The clinical symptoms of *Rotavirus*ncomplete.(15-18)

Studies of the genotyping in different regions of the world have indicated that G1-G4 and G9 types are the most common G types detected in children with *Rotavirus* gastroenteritis.(13, 19, 20) But in recent years, other rare or uncommon *Rotavirus* G types, such as G5, G8, G10, G11 and G12, have been reported in many countries.(6,14,21-24) In other diarrheal diseases, improvement of hygiene and sanitation may reduce incidences, but these measures are unlikely to be sufficient for *Rotavirus* control. Regarding to the high burden of *Rotavirus* infection, an effective *Rotavirus* vaccine program will reduce the morbidity associated with severe *Rotavirus* diarrhea. Before that program can be implemented, information is needed on the current burden of *Rotavirus* disease and the distribution and frequency of *Rotavirus* strains circulating in different regions of the country. The objectives of this study were to describe epidemiology of *Rotavirus* disease and determine the G types of *Rotavirus* circulating in children aged <5 years old with acute gastroenteritis in Shiraz, Iran.

## Materials and Methods

### Specimen collection

In this study a verbal consent was taken from either parent of the enrolled child prior to the interview and collection of stool samples. From December 2007 to November 2008, a total of 138 stool specimens were collected during the course of treatment from children under 5 years of age who were hospitalized with acute gastroenteritis in Shahid Dastgheib and Nemazee hospitals in Shiraz, Southern Iran. All the fecal specimens were transported to the infectious disease unit laboratory and stored at -70°C until the time of assay. All samples underwent only one cycle of thawing and freezing prior to characterization. A standard structured questionnaire was used to obtain the information regarding the age, sex, duration of hospital stay, severity of clinical symptoms and type of feeding (as breast/bottle feeding) for each case.

### *Rotavirus* detection 

All samples were screened for group A *Rotavirus* by enzyme immunoassay (EIA) (*Rotavirus* Ag ELISA, DRG, Germany), according to the manufacturer's instructions. 

### Viral RNA extraction 

Genomic RNA was extracted with a commercially available mixture of phenol and guanidine thiocyanate (RNX-Plus kit, CinnaGen, Tehran, Iran). Isolation of whole RNA was performed according to the manufacture’s protocol. Briefly, 500 µl RNX-Plus solution was mixed with a 20% stool suspension in phosphate buffer saline (PBS) at a pH of 7.2 at a volume ratio of 3 to 1. After complete dissociation of nucleoprotein complexes, chloroform was added to the mixture followed by vigorous shaking and centrifugation at 12,000 g for 15 min at 4°C. The upper aqueous phase was transferred to a fresh tube and the RNA precipitated by mixing it with isopropanol. The supernatant was removed and the RNA pellet was washed once with 75% ethanol. The RNA pellet was then briefly air dried and dissolved in diethyl pyrocarbonate (DEPC) treated water.

### Reverse transcription-polymerase chain reaction

Briefly, 5 µl of dsRNA was added to a mix of DMSO, 5X RT buffer, dNTPs, primers Beg9, End9,(25) and DW, denatured at 97°C for 5 min. Then RT enzyme and RNase inhibitor were added to make a final volume of 20 µl. The RT-PCR reaction was carried out for 60 min at 42°C to produce the complementary (cDNA) used for PCR amplification *Rotavirus*.

### Nested multiplex PCR for G genotyping

The G-typing was performed according to *Rotavirus* detection and typing protocol provided by WHO.(25) Briefly, the first round VP7 consensus PCR was carried out with 10 μl of cDNA in 40 μl of the VP7 reagent mixtures. The cycling parameters used were: 30 cycles at 94°C for 1 min, 42°C for 2 min, 72°C for 2 min, and a final extension at 72°C for 5 min. The second round VP7 multiplex PCR was carried out with 5 μl of first round VP7 amplicons in 40 μl of the second round VP7 reagent mixtures. Cycling was done with 20 cycles of the same cycling profile as the first reaction. The PCR mixtures contained 10x PCR buffer, MgCl2 (50 mM), deoxynucleoside triphosphates (10 mM), primers (10 pmol), and Taq DNA polymerase (1U). The amplified product was visualized by gel electrophoresis using 2% agarose gel containing ethidium bromide (10 μg/mL). The 100 bp DNA ladder (GeneRuler™, Fermentas life science) was used as a molecular weight standard. Primer sequences are shown in Table 1.(25)

**Table 1:** Primer sequences and positions used for genotyping of VP7 gene in Rotavirus strains. (25)

Primer	Sequence (5’→3’)	Position	Type
Beg9	GGC TTT AAA AGA GAG AAT TTC CGT CTG G	nt 1-28	-
End9	GGT CAC ATC ATA CAA TTC TAA TCT AAG	nt 1062-1036	-
aBT1	CAA GTA CTC AAA TCA ATG ATG G	nt 314-335	G1
aCT2	CAA TGA TAT TAA CAC ATT TTC TGT G	nt 411-435	G2
aET3	CGT TTG AAG AAG TTG CAA CAG	nt 689-709	G3
aDT4	CGT TTC TGG TGA GGA GTT G	nt 480-498	G4
aAT8	GTC ACA CCA TTT GTA AAT TCG	nt 178-198	G8
aFT9	** CTA GAT GTA ACT ACA ACT AC**	** nt 757-776**	** G9**

### Data analysis 

Data was statistically analyzed by SPSS version 15 (SPSS Inc., Chicago, IL, USA). Statistical analysis χ2 test was used to analyze the data obtained to the age group, sex, G types and seasonal distribution of the group A *Rotavirus* and also type of feeding. Fisher’s exact test was used to analyze the clinical symptoms. P value <0.05 was considered statistically significant.

## Results

### *Rotavirus* detection

A total of 48 (34.78%) diarrheic fecal specimens were confirmed as *Rotavirus* positive using EIA assay.

### *Rotavirus* and demographic data

**Figure 1:**
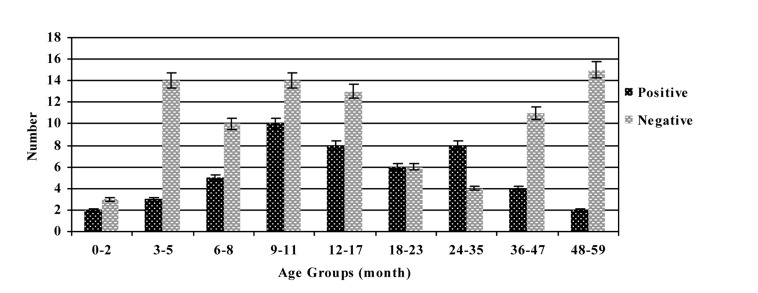
Age distribution of human Rotaviruses from December 2007 to November 2008

**Figure 2:**
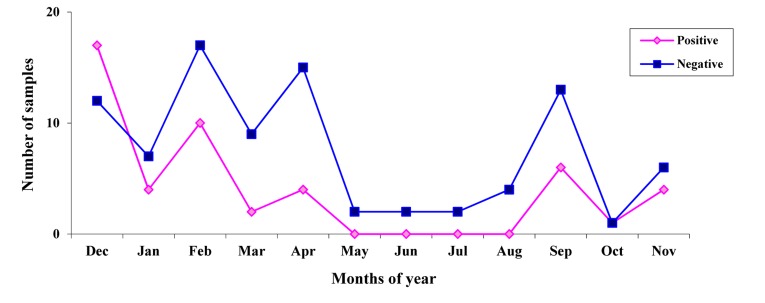
Monthly distribution of human Rotaviruses from December 2007 to November 2008

More males (68.75%) were infected than females (31.25%), and the male-to-female ratio of *Rotavirus* infection was 2.2:1 (P= 0.026). The age of infected children ranged from 1 to 59 months (Figure 1), and the median age was 7.9 months. Children less than 24 months of age accounted for 70.83% of the overall *Rotavirus*-positive cases with those between 9 and 11 months of age being the most affected (P= 0.052). The survey of clinical symptoms in *Rotavirus* gastroenteritis cases showed that children with infection had diarrhea (97.92%), vomiting (77.08%), fever (52.08%) and convulsion (6.25%). Also there was a relationship between *Rotavirus* infection diarrhea (P= 0.001) and convulsion (P= 0.049) symptoms. According to the season distribution, the highest prevalence of infection was identified in autumn (45.83%), followed by winter (33.34%),

Summer (12.50%) and spring (8.33%), respectively (P= 0.012). *Rotavirus* was detected continuously in the 8-month period lasting from September to April. *Rotavirus* was detected most frequently in December–February. The presence of *Rotavirus* remained low in May–August in which no G type was detected (P= 0.199) (Figure 2). Overall, 62.50 % of the children with acute gastroenteritis were not breastfed and 37.50 % were breastfeeding at the time of presentation of *Rotavirus* infection (P= 0.236). 

### *Rotavirus* genotyping

Genotyping was performed on 48 *Rotavirus* positive stool samples by using Nested RT-PCR (Figure 3). The most common circulating G type in the population under surveillance was mixed types, that being identified in 16 strains out of 48 samples (33.34%), followed by non-typeable (15/48, 31.25%), G4 (13/48, 27.08%), G1 (3/48, 6.25%) and G2 (1/48, 2.08%), respectively. The most prevalent mixed G types were G1/G4 (37.50%), G1/G8 (25%), G1/G3 (18.75%), G2/G8 (12.50%) and G3/G4 (6.25%), consecutively. The G types G3, G8 and G9 were not individually detected during the study. The most frequent detected type was mixed G types in females (53.33%) and G4 in males (33.33%) (P= 0.169). The most prevalent *Rotavirus* type reported was non-typeable and mixed infection (each 50%) in spring, non-typeable and G1 (each 33.33%) in summer, non-typeable and G4 in the autumn (each 36.36%) and mixed infection in the winter (50%) seasons (P= 0.049)

## Discussion

*Rotavirus* is a significant cause of diarrhea in developed and under developed countries in children under five years of age, and it is essential to determine the circulating genotypes and their temporal and geographical variations. From 2007 to 2008, constant surveillance of *Rotavirus* diarrhea in Shiraz, Iran, showed a prevalence of 34.78% in children less than 5 years of age with gastroenteritis. This result is comparable to the disease burden of *Rotavirus* seen in other studies in Iran and different countries which has shown to be between 13 to 40% of all cases of gastroenteritis. (10,12,14,16-18) Extensive studies in different regions of the world have indicated that in temperate climates, *Rotavirus* infection occurs predominantly during the cooler months.(9,26,27) On the other hand, seasonal patterns in tropical climates have shown rates of *Rotavirus* diarrhea throughout the year with seasonal trends that are less are less pronounced.(6,9,12,20,21) This study demonstrated that there was a significant correlation between the seasonal distribution and *Rotavirus*-positive cases. *Rotavirus* gastroenteritis occurred throughout the year, with more cases occurring in the winter with a seasonal peak observed in the months of December to February. These findings are similar to those reported in countries with temperate climates such as: Iran,(15,16) China,(26) Burkina Faso,(28) and Spain.(29) Similar surveillances for prolonged time period are needed in order to ascertain accurately the seasonality associated with *Rotavirus* infection in the studied area. The occurrence of the group A *Rotavirus* was cumulatively observed in the first 24 months of life (70.83%) more than in the older age groups, as was found in previous investigations.(11,16,26,28,30) *Rotavirus* age distribution was related to the peak incidence of infection, decline in maternal antibodies, and immaturity of new passive immune responses. The high frequency of *Rotavirus* gastroenteritis in this age group highlights the need for a vaccine to offer optimal protection against acute *Rotavirus* infection in children <2 years old. During this study, diarrhea was the symptom most commonly reported that is associated with *Rotavirus* infection, followed by vomiting, fever and convulsion. These findings are in keeping with studies conducted in Iran and other countries.(10,28,30-33) In the present study, evaluation of the breastfeeding status of infants less than 12 months of age with severe diarrhea showed that *Rotavirus*-positive cases were infrequent among those being breastfed at the time of acute gastroenteritis. This result suggests that breastfeeding may be a protective factor against *Rotavirus* infection, as reported in other studies.(28,30,31) Worldwide genetic diversity of circulating *Rotavirus* strains is associated with the presentation of new emerging strains, causing variability in the geographical distribution of the virus. The most common circulating G type in our study was mixed types with two different *Rotavirus* G types. The proportion of mixed infections (33.34%) reported in this study is substantially lower than what was reported in Guinea-Bissau (59%),(34) and Iran (60%),(35) and higher than studies conducted in Ireland,(36) Indonesia,(37) Africa,(38) India,(39) with the prevalence of 28.5%, 23%, 21.4% and 21%, respectively. In the current survey, we documented the first case of G1/G3 mixed infection in Iran. This G type has been reported in young children with gastroenteritis in Mexico.(40) Some of the mixed types detected in the present investigation are similar with those identified in other studies.(10,20,38,40) The high prevalence of mixed infections may reflect the observed diversity of strains. Patients harboring multiple *Rotavirus*es may offer a unique environment for the re-assortment process, facilitating generation of novel *Rotavirus* strains and the maintenance of the observed diversity.(15,34,39) Separately, the high mixed infection with different *Rotavirus* strains may be explained by greater environmental contamination with *Rotavirus*, for example water resources, coupled with greater contact between children and the environment around them.(39) Therefore, the frequency of mixed infection of *Rotavirus*es and its effect on the development of *Rotavirus* vaccine should be thoroughly investigated. During this study, 31.25% of the evaluated samples were G untypeable that might be related to the presence of novel strains, the failure of the genotyping due to the presence of the other genes was not investigated in this survey; for example, rare G types such as G5, G6, G10, G12, and failure in RT-PCR technique.(25) Non-typeable *Rotavirus* strains are rarely reported in children with acute diarrhea in Iran and other countries.(15,16,19,37,41) In recent years G4 strain has been detected at relatively high frequency from Iran,(15,42) to Italy,(19) to South Korea,(32) and Brazil.(41) The present study revealed G4 type as the prevalent G type individually in 27.08% of all *Rotavirus*-positive cases. Numerous molecular epidemiological studies have shown that G1 is the most common circulating G type worldwide. (6,14,15,37,43) However the G1 type was observed only in 6.25% of all children with *Rotavirus* diarrhea. We detected the G2 type in the specimens of only 2.08% of patients with acute diarrhea, which is in contrast with studies conducted in Italy, Sierra Leone and India, which demonstrated that the G2 type is one of the most prevalent *Rotavirus* types.(10,19,20,44) In the current study, neither the G3 nor the G8 types were detected individually. These findings are distinct from those results observed in Sierra Leone,(10) Turkey,(13) Malawi,(21) and China,(45) where these G types have been identified as the most common types in children. In recent years there has been an increase in research of the importance of G9 type in many countries including Latin America,(12) Iran,(16) Albania,(19) Brazil,(37) Cuba,(46) and Tanzania.(47) However, the G9 type was not detected during the study. Investigations in developing and industrialized countries have demonstrated the need for new generations of *Rotavirus* vaccines to include G9 strains due to the increasing emergence of this type of group A *Rotavirus*. In conclusion this study provides information on the epidemiology and description of the *Rotavirus* G types circulating among Iranian children with acute diarrhea. Our results indicate that gastroenteritis caused by *Rotavirus* in the country is a significant health problem, particularly among children less than 2 years of age and during the cold season. These data will be useful for making an informed decision about the introduction of *Rotavirus* vaccine in Iran and provides a baseline data for future vaccine studies.
