# Corrigendum to: Burden and Typing of Rotavirus Group A in Children with Acute Gastroenteritis in Shiraz, Southern Iran [published in Iran Red Cres Med J. 2012;14(9):531-540]

**DOI:** 10.5812/ircmj.9562

**Published:** 2013-02-05

This corrigendum/addendum supplies corrected statements and proofs of an error by author.

Hear by, authors declare that the corrected authors list and affiliations are:

Akram Najafi ^1^, Mohammad Kargar ^1,*^, Tarlan Jafarpour ^1^

^1^ Department of Microbiology, Jahrom Branch, Islamic Azad University, Jahrom, IR Iran

Sincerely Yours

Mohammad Kargar

